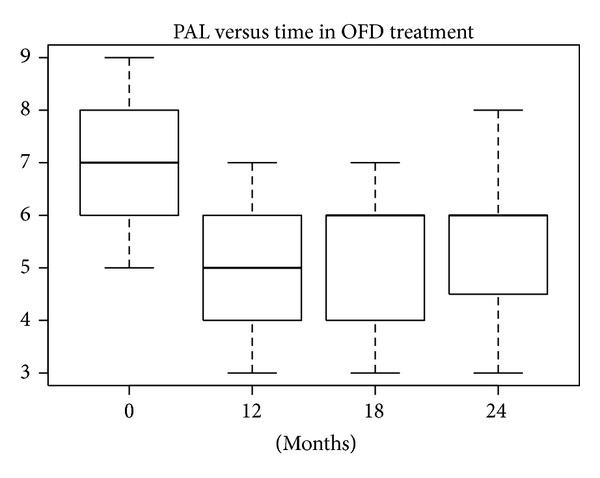# Clinical Evaluation of the Regenerative Potential of EMD and NanoHA in Periodontal Infrabony Defects: A 2-Year Follow-Up

**DOI:** 10.1155/2014/492725

**Published:** 2014-09-08

**Authors:** Andrea Pilloni, Matteo Saccucci, Gabriele Di Carlo, Blerina Zeza, Marco Ambrosca, Michele Paolantonio, Gilberto Sammartino, Claudio Mongardini, Antonella Polimeni

**Affiliations:** ^1^Department of Oral and Maxillofacial Science, Sapienza University of Rome, Via Caserta 6, 00161 Rome, Italy; ^2^Department of Medical, Oral and Biotechnological Sciences, University “G. d'Annunzio”, Chieti, Italy; ^3^Department of Oral Surgery, Faculty of Medicine, University Federico II, Naples, Italy

## Abstract

*Introduction.* The aim of this retrospective study was to compare the clinical efficacy of four different surgical techniques in promoting periodontal regeneration in patients with infrabony defects: open flap debridement, application of enamel matrix derivatives (EMD), nanohydroxyapatite (nanoHA) application, and combined nanoHA and EMD application. Probing attachment level (PAL), pocket depth (PD), and position of gingival margin at completion of therapy (REC) were measured. *Materials and Methods.* Data were collected from 64 healthy patients (34 women and 30 men, mean age 37,7 years). Clinical indices were measured by a calibrated examiner at baseline and at 12, 18, and 24 months. The values obtained for each treatment were compared using nonparametric tests. *Results.* All treatments resulted in a tendency toward PD reduction over time, with improvements in REC and PAL. The differences in PD, REC, and PAL values at baseline compared with values after 12, 18, and 24 months were statistically significant for all treatments. Statistically significant differences in PAL and PD were detected between nanoHA and nanoHA + EMD at 12, 18, and 24 months. *Conclusion.* In this study, EMD and nanoHA used together in patients with infrabony periodontal lesions had better clinical efficacy than nanoHA alone, EMD alone, or open flap debridement.

## 1. Introduction

In the past three decades periodontal regenerative treatment has received increasing attention as an alternative to tooth extraction in patients with periodontal disease. Implant insertion, although highly predictable, has been shown to be less predictable than saving a periodontally compromised tooth with regenerative therapy [1]. Given the proper conditions for optimal wound healing (wound stability, adequate space, and healing by primary intention), the periodontal tissues are capable of significant regeneration [2]. Unfortunately, systemic and local factors can interfere with these conditions, making periodontal regeneration difficult without the use of grafting biomaterials, biologics, and devices for periodontal regeneration [2]. Biomaterials in periodontal regeneration, as for bone reconstruction around implants, are of different sources [3, 4]. The ideal material for periodontal wound healing and regeneration would combine biologics with an easy-to-use, moldable, space-providing, biocompatible, bioadhesive, porous, and biodegradable matrix for local applications [2]. The simultaneous use of enamel matrix derivative (EMD) and nanohydroxyapatite (nanoHA) seems to fulfill the aforementioned criteria for an ideal combination. The* in vitro* combination has shown promising results, with each material stimulating periodontal fibroblasts differently, enhancing the potential of each [5]. Amelogenins, present in EMD, are extracellular matrix proteins that induce the formation of acellular cementum when absorbed on the root surface [6] and that stimulate the proliferation and differentiation of periodontal fibroblasts and osteoblasts [7, 8]. The role of amelogenins in periodontal regeneration is primarily related to regeneration of the periodontal ligament and cementum. In contrast, nanoHA is an alloplastic material chemically similar to the inorganic component of bone matrix and is known for its osteoinductive and osteoconductive properties in alveolar bone regeneration [9]. The clinical relevance of EMD is supported by its long-term outcome stability in studies with 10–15 years of follow-up [10]. In contrast, the literature on nanoHA remains scarce and contradictory. In 2008, Kasaj et al. [11] reported significant clinical improvement in patients treated with nanoHA compared with open debridement. However, in 2013, Horváth et al. [12] could not confirm those findings. In 2014, Al Machot et al. [13] reported that EMD and nanoHA powder performed similarly and resulted in significant bone filling and clinical improvement. The aim of the present study was to compare the clinical efficacy of EMD and nanoHA applied individually and in combination, using open flap debridement (OFD) as a control.

## 2. Materials and Methods

### 2.1. Study Design and Population

This retrospective study was conducted in the Department of Oral and Maxillofacial Science, Sapienza University of Rome, Italy. Data were collected between 2009 and 2012. Sixty-four generally healthy patients (34 women and 30 men; mean age 37,7 years) were screened for inclusion. Study inclusion criteria were as follows:

- age over 18 years;
- systemic health: lack of acute or chronic condition that would contraindicate oral surgery;
- bone defect characteristics: a single 1-, 2-, or 3-wall infrabony defect more than 3 mm deep on radiographs, with pocket depth (PD) ≥5 mm;
- tooth condition: periodontally, endodontically, and prosthetically healthy tooth.

Patients were excluded for the following reasons:

- pregnancy or lactation;
- taking medications that could interfere with the healing of periodontal tissues;
- furcation involvement;
- overhanging restorations;
- teeth with grade 2 or higher mobility;
- teeth with endodontic lesions.

**Figure 1:**
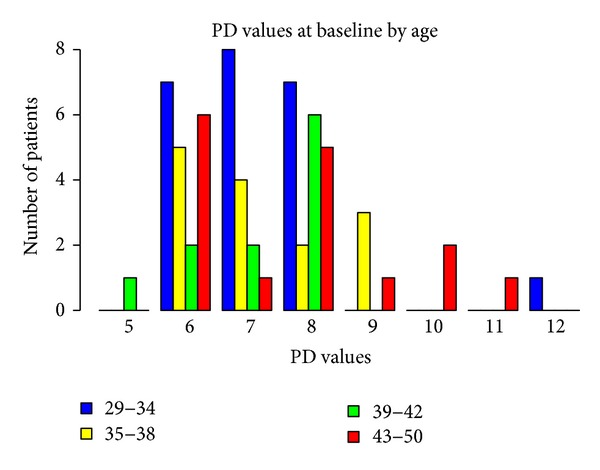


**Figure 2:**
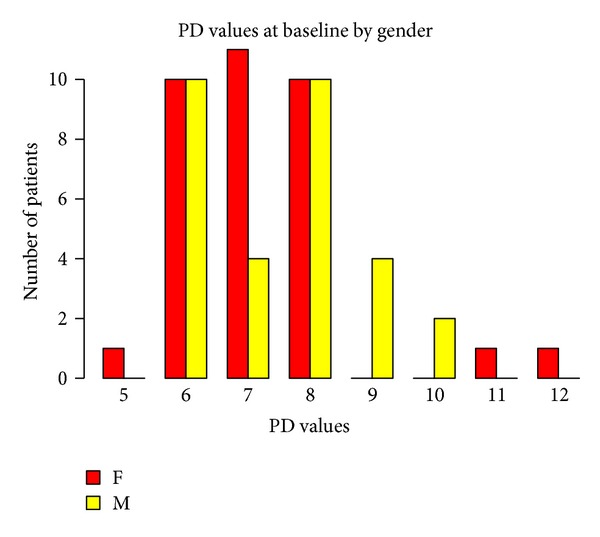


**Figure 3:**
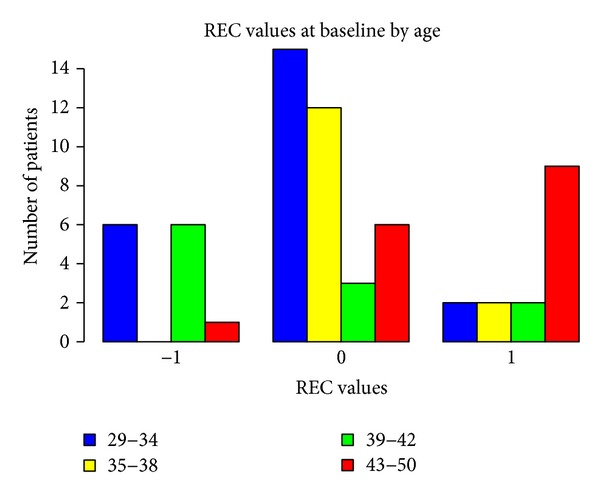


**Figure 4:**
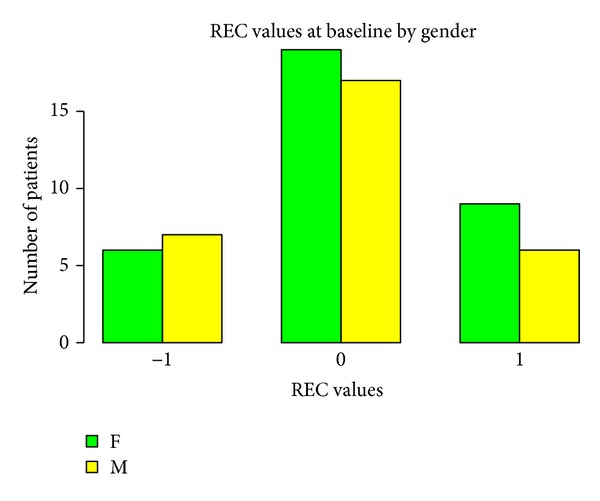


**Figure 5:**
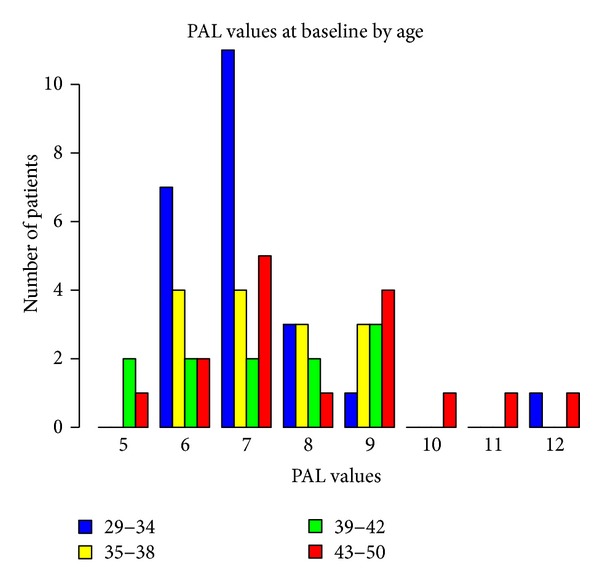


**Figure 6:**
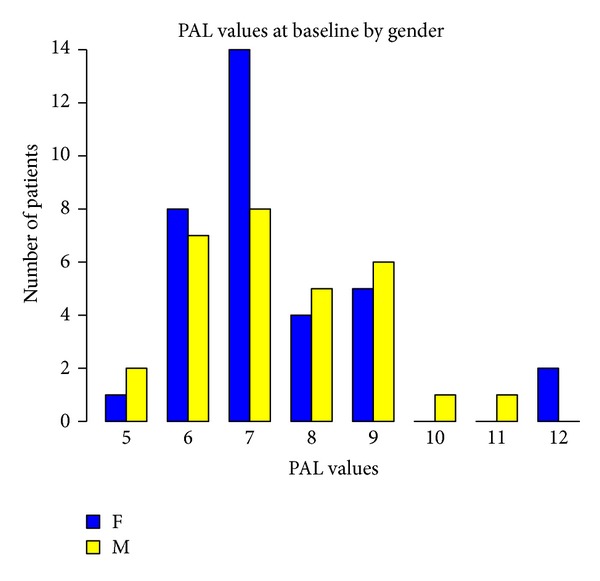


Baseline measurements of probing attachment level (PAL), position of gingival margin at completion of therapy (REC), and PD, according to gender and age, are shown in Figures 1, 2, 3, 4, 5, and 6. Chronic periodontitis was diagnosed in all patients, because the location of defects and number of teeth affected did not meet the criteria of aggressive periodontitis, even in those younger than 30 years of age. Each patient participating in this study signed a consent form approved by the Ethical Committee of the Faculty of Medicine, G. D'Annunzio University, Chieti, Italy. The study protocol was performed in accordance with the Declaration of Helsinki of 1975, revised in Tokyo in 2004.

### 2.2. Treatments

Patients were randomly allocated to one of the following surgical treatment groups:

- open flap debridement;
- EMD application (Emdogain Straumann, Basel, Switzerland);
- nanoHA (NeoActive Ghimas, Casalecchio di Reno, Italy);
- Sandwich technique combining nanoHA and EMD.

All patients underwent the same presurgical and surgical procedure, differing only in the material used for bone regeneration.

### 2.3. Presurgical Treatment

All patients underwent phase I therapy with revaluation within 3 months. If the full-mouth plaque score and full-mouth bleeding score were under 15% at recheck, the patient entered the surgical protocol. Otherwise, an additional motivation and professional cleaning phase was performed.

### 2.4. Periodontal Surgery

After local anesthesia, intrasulcular incisions were made, in conjunction with vertical releasing incisions if needed for bone defect exposure. A full-thickness flap was then prepared, the bone defect exposed, and granulation tissue removed with manual and ultrasonic root planing instruments. The area of the infrabony defect was rinsed with saline, dried, and treated according to treatment group allocation:

- chemical root conditioning (EDTA, 24%) before application of EMD to the root surface;
- application of adequate nanoHA powder to fill the defect and maintain root surface area sufficient for its biological width to be reestablished;
- chemical root conditioning (EDTA, 24%), EMD application, bone defect filling with nanoHA, and layer of EMD over of the bone substitute, using the sandwich technique;
- open flap debridement.

**Figure 7:**
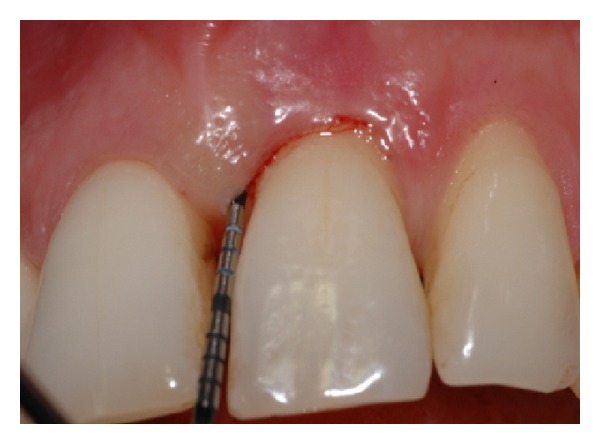
Probing at baseline showing PAL = 7 mm on the mesial aspect of tooth number 9.

**Figure 8:**
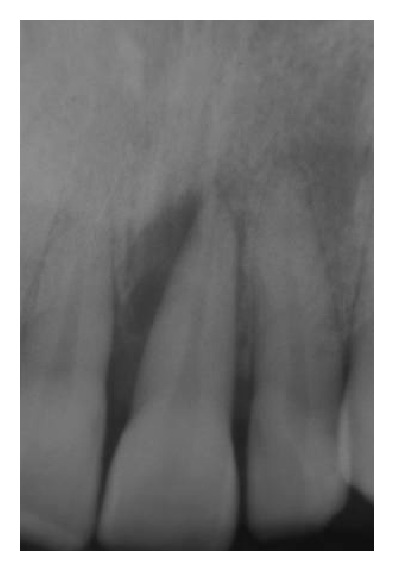
Periapical X-ray at baseline.

**Figure 9:**
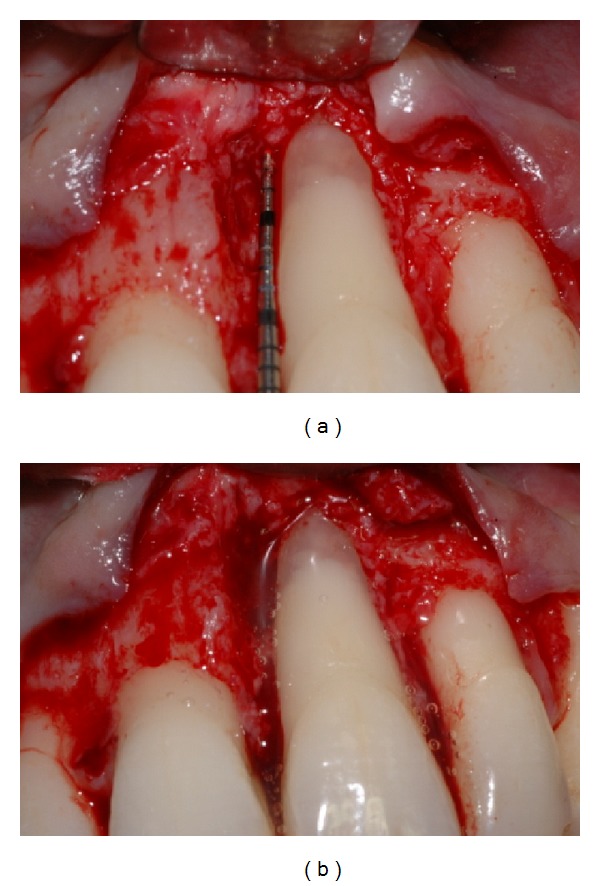
(a) Root surface appearance after flap reflection and degranulation. (b) EMD applied on root surface.

**Figure 10:**
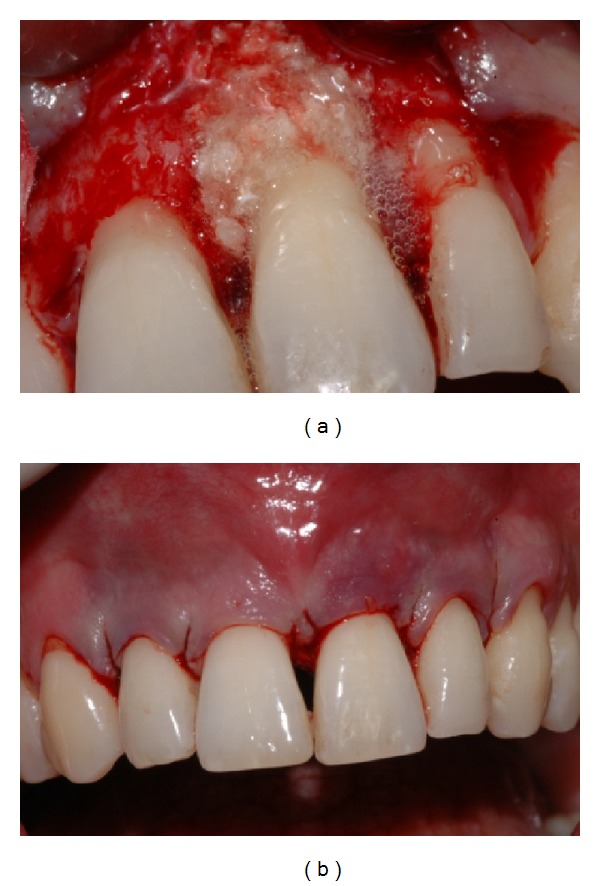
(a) NanoHA + EMD application on root surface using sandwich technique. (b) Sutured flap.

**Figure 11:**
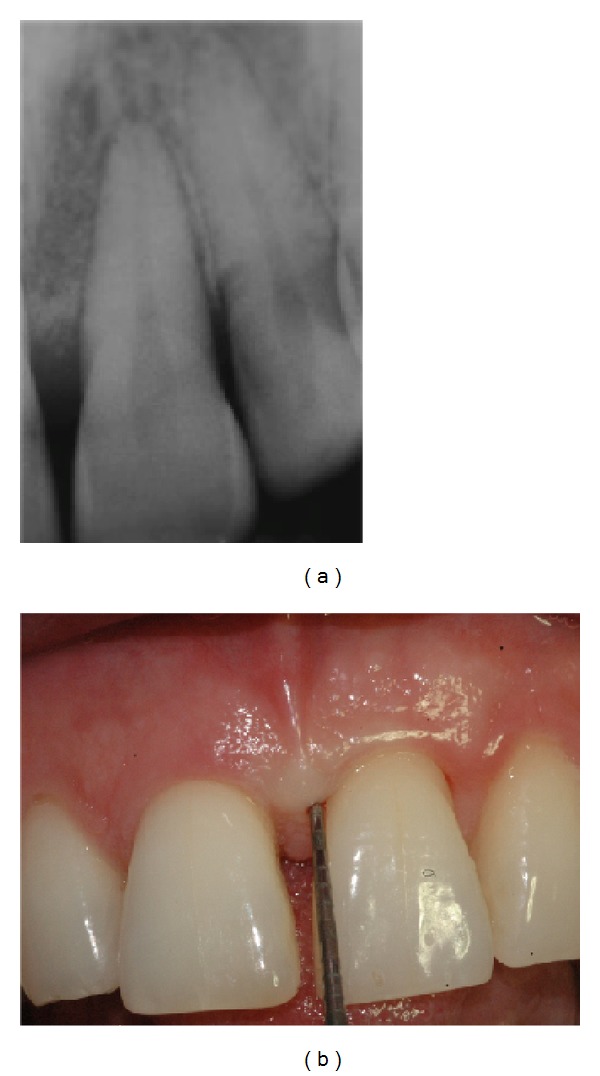
(a) X-ray at 24 months. (b) Clinical aspect showing attachment gain.

Gingival flaps were closed with 5–0 nylon suture in a simple interrupted pattern (Figures 7, 8, 9(a), 9(b), 10(a), 10(b), 11(a), and 10(b)).

### 2.5. Postsurgical Treatment

Patients received a prescription for amoxicillin, 1 g every 12 h for 5 days, and for ibuprofen, 600 mg every 12 h for 3 days. The use of synthetic ice in 5-minute intervals for the first 30 minutes was prescribed immediately after completion of surgery. All patients used 0.12% chlorhexidine rinse for 1 min twice daily, not within 1 hour of tooth brushing. Patients were examined each week for the first month and each month during the first year. Frequency of rechecks after the first year was customized based on the patient's plaque control and level of compliance. During recheck appointments patients underwent professional oral hygiene treatment.

### 2.6. Examiner and Surgeon

One individual was selected to make clinical measurements and a second to perform surgical treatments. Surgical treatments were performed by a highly experienced surgeon (AP).

### 2.7. Clinical Parameters

Patients were evaluated at baseline and at 12, 18, and 24 months after regenerative therapy by the above-mentioned calibrated dental hygienist. Clinical parameters measured at each time point included PD, REC, and PAL. All measurements were recorded using a standard periodontal probe (PCP-15, UNC) at six sites per tooth: mesiobuccal, midbuccal, distobuccal, mesiolingual, midlingual, and distolingual. The highest value obtained for PD and PAL and the lowest for REC were considered for statistical analysis. As suggested by Kasaj et al. [11], the cementoenamel junction or restoration margin was used as the fixed reference point.

### 2.8. Statistical Analysis

Statistical analyses were performed with R software, version 3.1.0. Clinical parameters were described as minimum, 1st quartile, median, 3rd quartile, maximum, mean, and standard deviation and were calculated by treatment (OFD, nanoHA, EMD, and nanoHA + EMD), time (baseline, 12 months, 18 months, and 24 months), site (mesiovestibular, distovestibular), and position (anterior, posterior). First, we evaluated differences in PAL, REC, and PD among patients assigned to each treatment. We accepted the hypothesis of equal location parameters at 5%. To test the efficacy of each treatment after 12, 18, and 24 months, the null hypothesis was that the mean of PD, REC, and PAL in each treatment group was the same at baseline and after 12, 18, and 24 months. The normal distribution of data within each group was confirmed with the Shapiro-Wilks test (SW test) [14]. If the hypothesis of normal distribution of the data was confirmed, comparisons among groups were made with a *t*-test or analysis of variance. If the hypothesis of normal distribution was rejected, then comparisons among groups were performed with Wilcoxon's test for paired data (W test) [15]. Values of *P* < 0.05 were considered significant. Median and mean differences in the location parameters for PD, REC, and PAL among the four treatment groups at 12, 18, and 24 months were tested for statistical significance. If the hypothesis of normality in the data was rejected according to the SW test, the nonparametric Kruskal-Wallis test (KW test) was performed in place of analysis of variance. If the null hypothesis where the median and mean location parameters were the same in each group was rejected, Siegel and Castellan's post hoc multiple comparison was performed at 5% significance level.

## 3. Results

The group that received EMD+HA showed greater mean reduction in PD (5.75 mm) and improvement in gingival recession compared with the EMD-only group. The group that received nanoHA alone had the poorest clinical results, although they did improve from baseline. All treatments resulted in PD reduction over time, with improvements in REC and PAL.

### 3.1. Differences between Baseline and Follow-Ups

#### 3.1.1. PD Index

**Figure 12:**
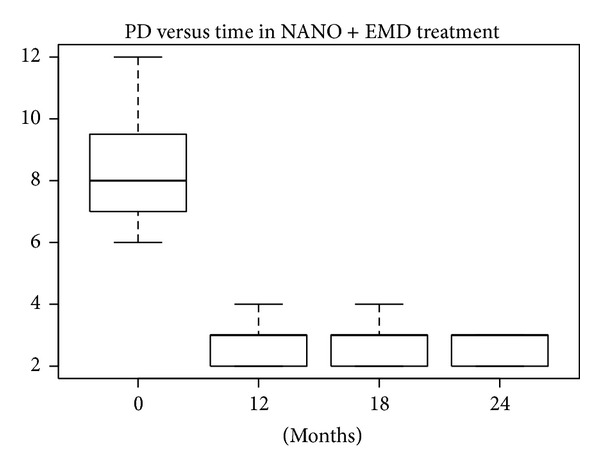


**Figure 13:**
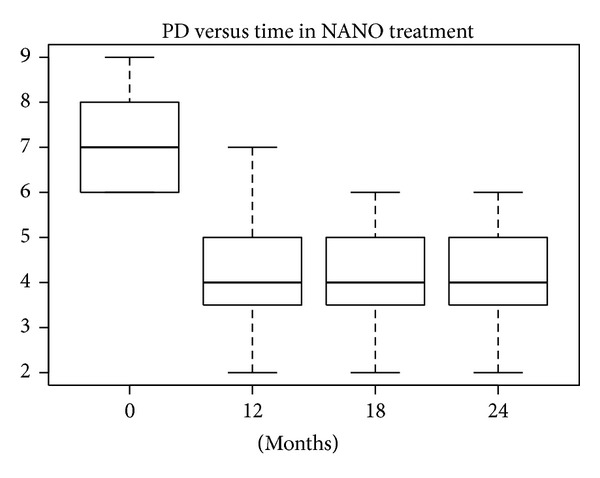


**Figure 14:**
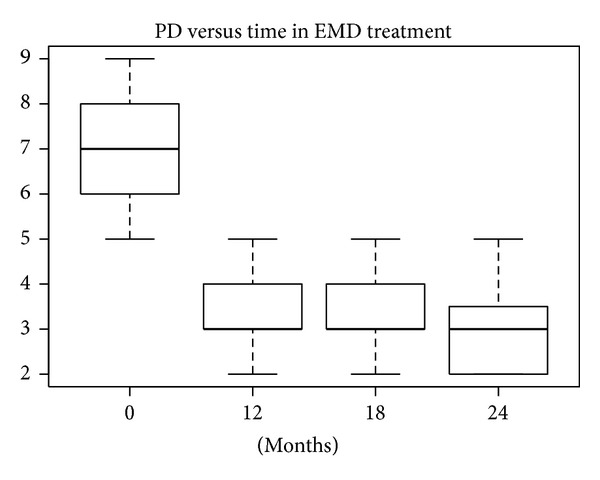


**Figure 15:**
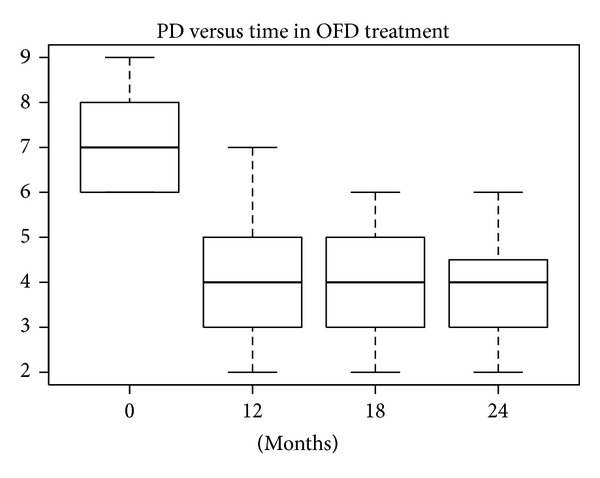


According to the SW test, the hypothesis of normality could be confirmed only for baseline data in the nanoHA + EMD group (*P* = 0.26). In the other groups the hypothesis was rejected, because *P* values were near 5% or much lower. The differences between PD levels at baseline and after 12, 18, and 24 months were statistically significant (W test, *P* < 0.01) for all treatments. The reductions in median and mean PD levels shown in Figures 12, 13, 14, and 15 are significant.

#### 3.1.2. REC Index

**Figure 16:**
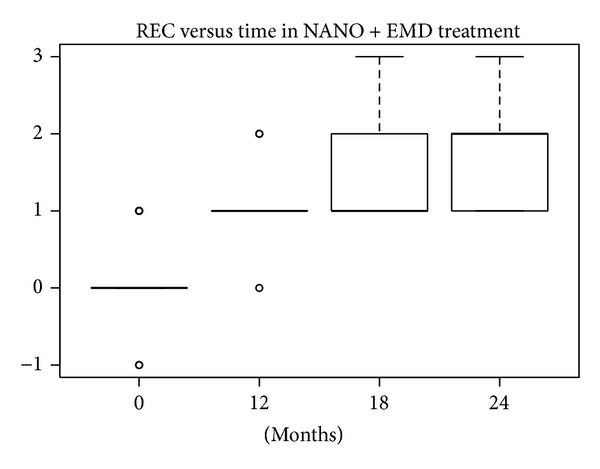


**Figure 17:**
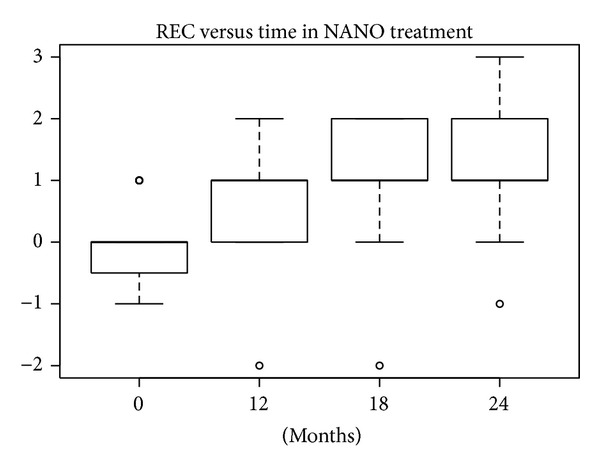


**Figure 18:**
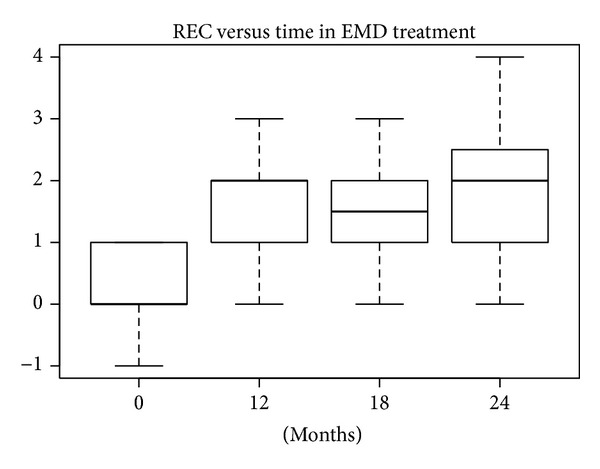


**Figure 19:**
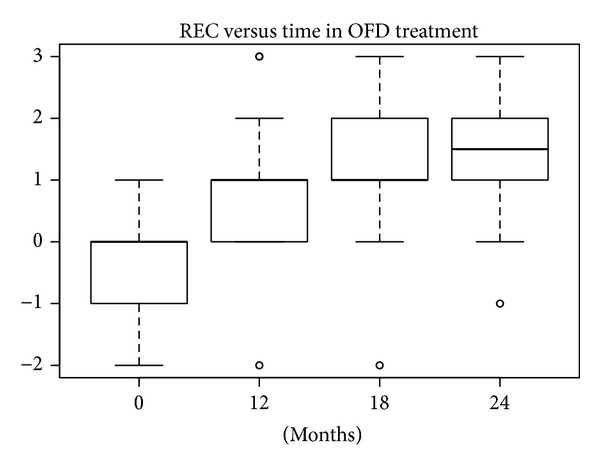


According to the SW test, the hypothesis of normality could be accepted at 5% significance level only for nanoHA at 12 months and for EMD at 18 and 24 months. In the other groups the hypothesis was rejected, because *P* values were lower or much lower than 5%. The differences in REC levels at baseline compared with levels after 12, 18, and 24 months were statistically significant (W test, *P* < 0.01) for all treatments. The reductions in median and mean REC levels shown in Figures 16, 17, 18, and 19 can be considered significant.

#### 3.1.3. PAL Index

**Figure 20:**
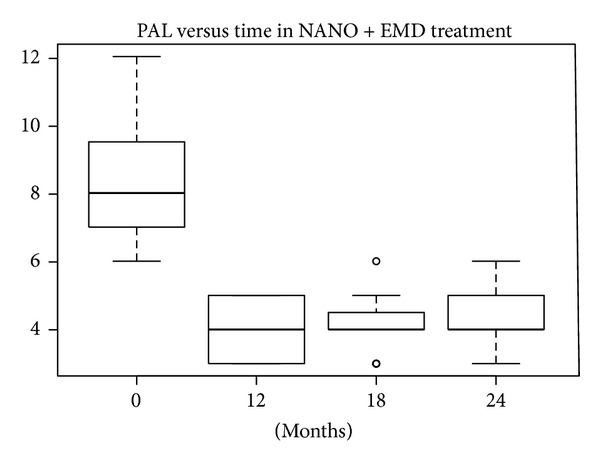


**Figure 21:**
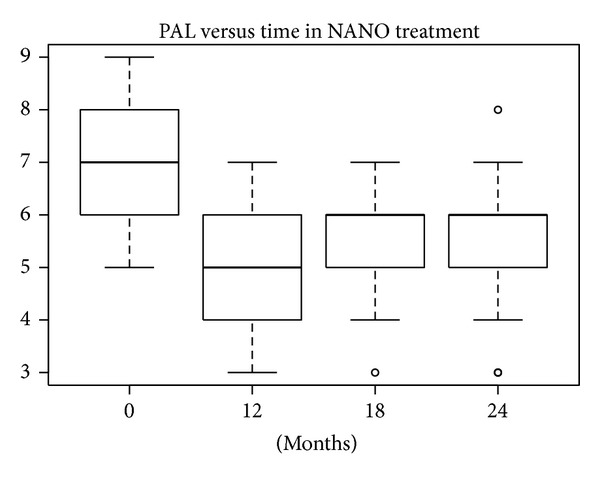


**Figure 22:**
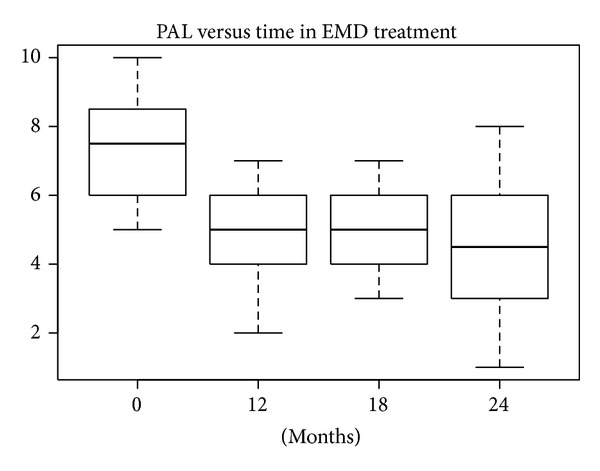


According to the SW test, the hypothesis of normality could be accepted at 5% significance level only in the EMD treatment group, so both the *t*-test and W test were performed. In the other treatment groups the hypothesis of normality was rejected (*P* values lower than 5%). The differences in REC levels at baseline compared with levels after 12, 18, and 24 months were statistically significant at 10% for defects treated with nanoHA after 18 and 24 months and at 5% for the other treatments. The median and mean reductions in PAL levels shown in Figures 20, 21, 22, and 23 were significant.

### 3.2. Differences among Treatments after 12 Months

The null hypothesis where location parameters were the same among treatments was rejected for PD, REC, and PAL (*P* < 0.03). For PD, post hoc multiple comparison at 5% showed statistically significant differences between nanoHA and nanoHA + EMD (observed difference (obs diff) = 33.01, critical difference (crit diff) = 21.12) and between nanoHA + EMD and OFD (obs diff = 24.29, crit diff = 21.12). For REC, post hoc multiple comparison at 5% showed statistically significant differences between EMD and nanoHA (obs diff = 22.05, crit diff = 21.12). Post hoc multiple comparison for PAL at 5% showed statistically significant differences between nanoHA and nanoHA + EMD (obs diff = 24.33, crit diff = 21.12) in favor of nanoHA + EMD.

### 3.3. Differences among Treatments after 18 Months

The null hypothesis where location parameters were the same among treatments was accepted for REC (*P* = 0.53) and rejected for PD and PAL (*P* < 0.01). Median and mean location parameters for REC were not statistically different among treatments; parameters were significantly different for PD and PAL (*P* < 0.03).

For PD, post hoc multiple comparison at 5% showed statistically significant differences between nanoHA and nanoHA + EMD (obs diff = 31.38, crit diff = 21.12) and between nanoHA + EMD and OFD (obs diff = 24.13, crit diff = 21.12). These were the same results as at the 12-month evaluation. For PAL, post hoc multiple comparison at 5% showed statistically significant differences between nanoHA and nanoHA + EMD (obs diff = 26.75, crit diff = 21.12) and between nanoHA + EMD and OFD (obs diff = 26.25, crit diff = 21.12).

### 3.4. Differences among Treatments after 24 Months

The null hypothesis that location parameters were the same among treatments was accepted for REC (*P* = 0.28) and rejected for PD and PAL (*P* < 0.02). Median and mean location parameters were not statistically different among treatments for REC, but they were different for PD and PAL. The same results were obtained as for the 18-month evaluation (*P* < 0.03).

For PD, post hoc multiple comparison at 5% showed statistically significant differences between EMD and nanoHA (obs diff = 22.27, crit diff = 21.12), between nanoHA and nanoHA + EMD (obs diff = 33.58, crit diff = 21.12), and between nanoHA + EMD and OFD (obs diff = 25.26, crit diff = 21.12). For PAL, post hoc multiple comparison at 5% showed statistically significant differences between nanoHA and nanoHA + EMD (obs diff = 21.55, crit diff = 21.12).

## 4. Discussion 

All four methods employed in this study fulfilled the main purposes of periodontal surgery: controlling periodontal infection and providing periodontal maintainable sites. Thus, reduction of PD was found in all four treatment groups. However, periodontal regeneration requires more than control of infection and gingival recession. Calculation of the clinical attachment level reflects the degree of regeneration obtained. In this study, gingival recession showed an improving trend over time in all treatment groups. Regeneration without significant gingival recession was statistically important for OFD, EMD, and nanoHA + EMD but not for nanoHA alone. NanoHA alone showed less beneficial clinical effect than removal of granulation tissue without use of defect-filling material. This was an unexpected result, because biomaterials, especially osteoconductive materials such as nanoHA, are supposed to provide support for soft tissues and to prevent their prolapse. However, the clinical observations in the present study are in accordance with the histological results of Horváth et al., showing the limited potential of nanoHA in promoting periodontal regeneration over a 7-month period [12]. This finding could be explained by the rationale of Susin and Wikesjö [2], who reported that most biomaterials interfere with rather than support periodontal regeneration because of their sequestration within connective tissues. Susin and Wikesjö [2] and Trombelli et al. [16] evaluated a minimally invasive surgical protocol as a stand-alone approach or in combination with membranes and hydroxyapatite-based biomaterials. No significant differences were observed between experimental groups; sites treated with a minimally invasive technique exhibited clinical attachment gain averaging 4.7 ± 2.5 mm, probing depth reduction of 5.3 ± 2.4 mm, and gingival recession of 0.4 ± 1.4 mm. Similarly, Cortellini and Tonetti [17] evaluated a minimally invasive surgical technique, alone and in conjunction with an EMD and a bovine bone-based biomaterial. No significant differences between treatments were observed; the minimally invasive surgical approach without additions achieved substantial clinical attachment gain of 4.1 ± 1.2 mm and radiographic bone fill of 77 ± 19%. In contrast, our results suggest a significant difference between nanoHA and nanoHA + EMD in terms of changes in PD and PAL indices, measured at each follow-up interval. Our results suggest that EMD plays a prominent role in promoting better results when used in conjunction with nanoHA, compared with nanoHA alone. This clinical outcome is supported by a 12-month follow-up study conducted by Kasaj et al. [11], who reported that EMD had advantages over nanoHA in patient comfort and adverse effects. Kasaj et al. [5] reported that EMD acts as a chemoattractant, while nanoHA paste is a synthetic extracellular matrix component in its coated form. Al Machot et al. [13] hypothesized a potential synergic effect of combining the materials with resulting possible beneficial effects on wound healing.

Avoiding the Hawthorne effect is supposed to provide a more realistic picture of how a treatment functions in everyday practice. However, retrospective treatment evaluations have limitations that could bias the results. However, this bias can be reduced by using multiple control groups as in the present study, which compared four treatment groups, and by the triple blinding of the operator, the patient, and the analyst. Moreover, restricting patient selection criteria reduced confounding factors. One important tool in analyzing clinical outcome from the patient's perspective is the visual analog scale, which can confirm or cast doubt on the effects of biomaterials and techniques as regenerative strategies [18]. Such evaluation could have been useful in our study; postoperative data, including patient perception, could have been matched to clinical outcome, in both short- and long-term evaluation. One limit of the present study is that clinical parameters alone were used to evaluate the regeneration process. Previous studies have associated these parameters with radiographic measurements to reduce possible biases. However, clinical and radiographic parameters cannot fully replace histological analysis to confirm the true regeneration of all periodontal tissues. Therefore, even using both sources of information could be considered insufficient. Because the purpose of treatment is controlling infection by eliminating periodontal pockets and at the same time preventing soft tissue collapse, clinical data can be considered a primary outcome in everyday clinical practice. The combined use of nanoHA and EMD seems to fulfill such clinical criteria.

## 5. Conclusions

In summary, within the limitations of this study, we found that EMD and nanoHA played a synergic role in promoting restoration of the tooth-supporting apparatus.

## Figures and Tables

**Figure 23:**